# Downregulation of lncRNA SBF2-AS1 inhibits hepatocellular carcinoma proliferation and migration by regulating the miR-361-5p/TGF-β1 signaling pathway

**DOI:** 10.18632/aging.203248

**Published:** 2021-08-02

**Authors:** Yan-Hui Wu, Bin Yu, Wei-Xun Chen, Xi Ai, Wei Zhang, Wei Dong, Ya-Jie Shao

**Affiliations:** 1 Hepatic Surgery Center, Tongji Hospital, Tongji Medical College, Huazhong University of Science and Technology, Wuhan 430030, China; 2 Department of General Surgery, The First Affiliated Hospital of Nanchang University, Nanchang 330006, China; 3 Department of Anesthesiology, Tongji Hospital, Tongji Medical College, Huazhong University of Science and Technology, Wuhan 430030, China

**Keywords:** hepatocellular carcinoma, lncRNA SBF2-AS1

## Abstract

SBF2-AS1 is an oncogenic long non-coding RNA (lncRNA). However, its role and mechanism in hepatocellular carcinoma (HCC) is still not completely clear. The HepG2, Hep3B, Bel-7402 and HL-7702 cell lines were used in our experiments. The CCK-8 kit and EdU staining were applied to detect cell viability and multiplication. The wound healing and Boyden chamber cell migration assays were employed to test the migration ability of cells. The levels of TGF-β1 mRNA, lncRNA SBF2-AS1, and miR-361-5p were assessed by real-time PCR. TGF-β1 protein levels were evaluated by western blotting. The direct interaction between miR-361-5p and TGF-β1 was determined by luciferase reporter assays. A xenograft mouse model (XMM) was established to comprehensively study the effect and mechanisms of lncRNA SBF2-AS1. lncRNA SBF2-AS1 concentration in HCC cells exceeded that in a normal hepatocyte cell line. The downregulation of lncRNA SBF2-AS1 upregulated miR-361-5p levels in HCC cells. And, miR-361-5p negatively regulate TGF-β1 expression in HCC cells. The suppression of miR-361-5p attenuated the influence of lncRNA SBF2-AS1 downregulation on the viability, proliferation, and migration capability of HCC cells. Further, the downregulation of lncRNA SBF2-AS1 inhibited neoplasm growth in an XMM of HCC. Simultaneously, miR-361-5p was upregulated and TGF-β1 was downregulated after lncRNA SBF2-AS1 knocked down. In conclusion, downregulation of lncRNA SBF2-AS1 inhibits HCC proliferation and migration through the regulation of the miR-361-5p/TGF-β1 signaling pathway.

## INTRODUCTION

Hepatocellular carcinoma (HCC) is considered one of the major causes of death worldwide, accounting for more than 600,000 deaths each year [1]. However, the diagnosis and treatment of HCC are still fraught with issues.

Non-coding RNAs are emerging as new diagnostic and therapeutic agents in various diseases. Recent studies have focused on long non-coding RNAs (lncRNAs) in HCC. lncRNAs are aberrantly expressed in HCC tissues. Using microarray analysis, 612 lncRNAs have been identified at different levels of HCC tissues [2]. Some lncRNAs are major factors in carcinogenesis and disease progression. This includes lncRNA homeobox A11 antisense (HOXA11-AS), which is upregulated in relevant cell lines and HCC tissues. The enhanced expression of this lncRNA can promote proliferation, invasion, and epithelial-mesenchymal transition (EMT) of HCC cells. Further, the downregulation of HOXA11-AS exerts an inhibitory effect on HCC [3]. Nrf2-activating lncRNA (Nrf2-lncRNA) is highly expressed in the liver and is associated with recurrence-free survival of HCC patients after surgery. Nrf2-lncRNA is also a regulatory lncRNA, which can promote Plk2 and p21^cip1^ translation by sponging microRNAs (miRNAs) [4]. lncRNA is a regulator of reprogramming (linc-ROR) and downregulates the level of TGF-β1 in HCC cells (HepG2-R and SMMC-7721-R). Moreover, the downregulation of linc-ROR attenuates the radioresistance of HCC cells [5]. However, the role and regulatory mechanism of most differentially expressed lncRNAs are still unknown.

SET-binding factor 2 antisense RNA1 (SBF2-AS1) is situated at the 11p15.1 locus. It is significantly upregulated in various malignancies, including breast cancer, lung adenocarcinoma, glioblastoma and acute myeloid leukemia [6–9]. In accordance with these findings, SBF2-AS1 is also elevated in tissues affected by HCC and is related to a poor HCC prognosis [10]. Our research goal was to further elucidate the underlying mechanisms of SBF2-AS1 in HCC.

## RESULTS

### Level of lncRNA SBF2-AS1 increased in HCC cells

**Figure 1:**
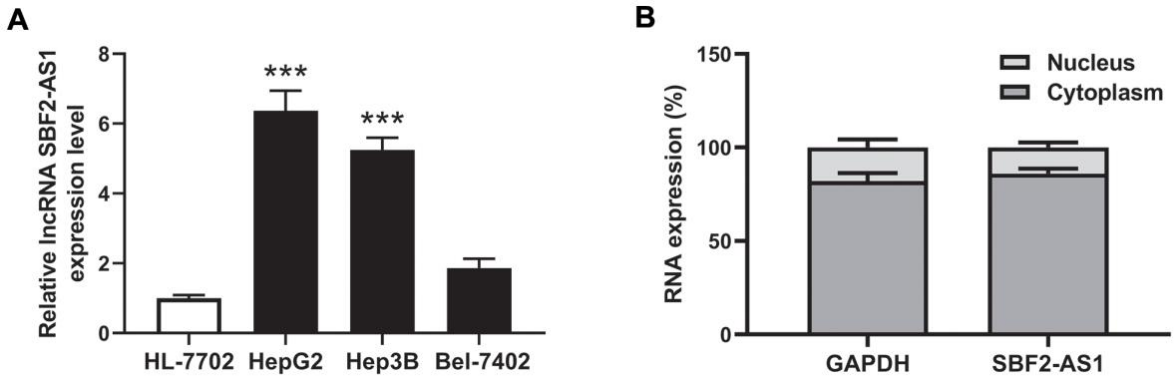
**Expression and location of lncRNA SBF2-AS1.** (**A**) Expression of lncRNA SBF2-AS1 in HCC and normal hepatocyte cells. (**B**) Location of lncRNA SBF2-AS1 in HepG2 cells. The data are expressed as mean ± SEM. *** P < 0.05 vs. HL-7702; n=3.

The level of lncRNA SBF2-AS1 in the human HCC cell lines, HepG2, Hep3B, Bel-7402 was detected, as well as in the regular hepatocyte cell line, HL-7702. Compared to normal hepatocyte HL-7702 cells (Figure 1A), the data revealed that the level of lncRNA SBF2-AS1 was higher in both the HepG2 and Hep3B HCC cell lines. No statistical significance was observed between Bel-7402 and HL-7702 (Figure 1A). Therefore, HepG2 and Hep3B was used in the following experiment.

The location of lncRNA SBF2-AS1 was predicted by iLoc-lncRNA (http://lin-group.cn/server/iLoc-LncRNA/pre.php). The prediction results showed that the subcellular location of lncRNA SBF2-AS1 is Cytoplasm, Cytosol. The probability score is 0.979693. To confirm the location of lncRNA SBF2-AS1, the subcellar fractions were separated in HepG2 cells. The results showed that lncRNA SBF2-AS1 was mainly distributed in the cytoplasm (Figure 1B).

### Downregulation of lncRNA SBF2-AS1 increased the level of miR-361-5p in HCC cells

**Figure 2:**
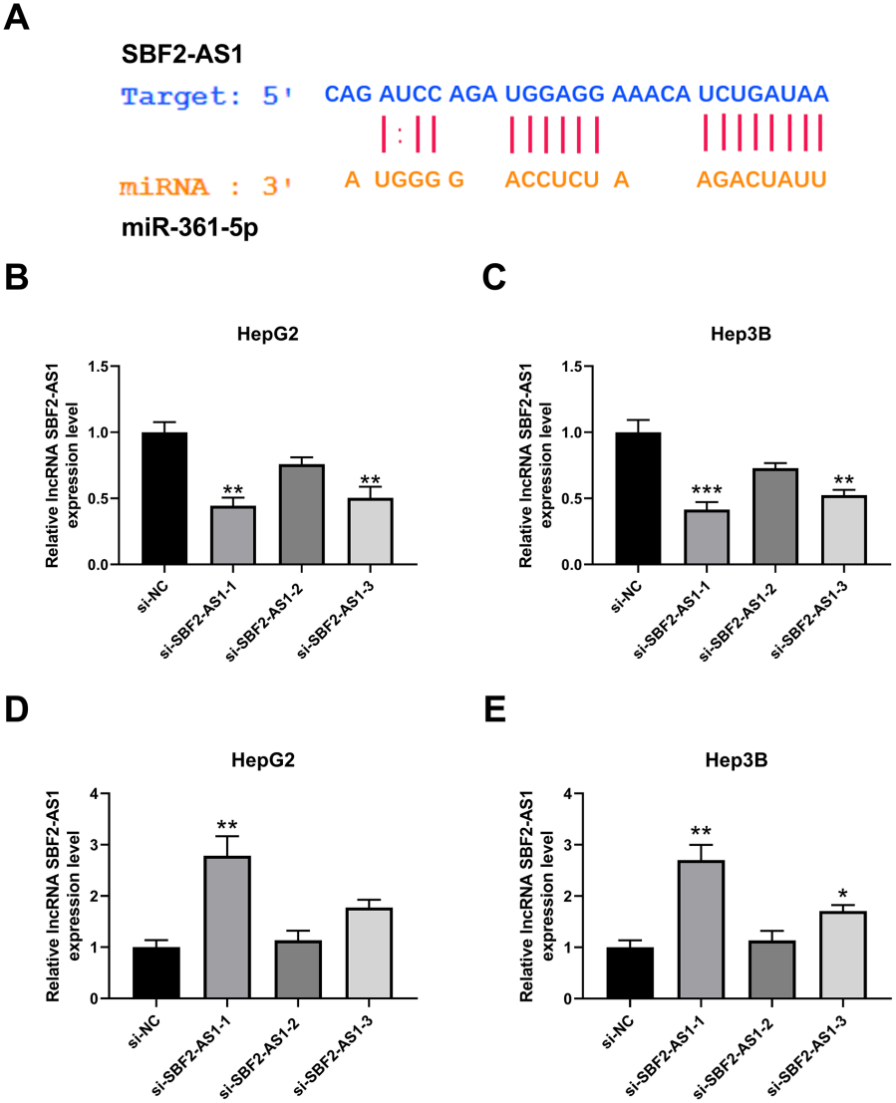
**Effect of lncRNA SBF2-AS1 on miR-361-5p.** (**A**) Prediction of a connection site of lncRNA SBF2-AS1 and miR-361-5p. (**B**) The influence of si-SBF2-AS1 on lncRNA SBF2-AS1 in HepG2 cells. (**C**) The influence of si-SBF2-AS1 on lncRNA SBF2-AS1 in Hep3B cells. (**D**) The effect of lncRNA SBF2-AS1 downregulation on miR-361-5p in HepG2 cells. (**E**) The influence of lncRNA SBF2-AS1 downregulation on miR-361-5p in Hep3B cells. The obtained data are expressed as mean ± SEM. * P < 0.05, ** P < 0.01 vs. si-NC; n=3.

To explore the potential mechanism underlying lncRNA SBF2-AS1, we predicted the miRNA binding sites with lncRNA SBF2-AS1 using ENCORI (http://starbase.sysu.edu.cn/index.php) [11]. The prediction results showed that miR-361-5p have binding sites with lncRNA SBF2-AS1 (Figure 2A). To establish the influence of lncRNA SBF2-AS1, small interfering RNAs were employed to downregulate the level of lncRNA SBF2-AS1. Three sequences (si-SBF2-AS1-1, si-SBF2-AS1-2 and si-SBF2-AS1-3) were designed, and the relevant efficiency was tested using real-time PCR. The results showed that both si-SBF2-AS1-1 and si-SBF2-AS1-3 could downregulate lncRNA SBF2-AS1 levels in HepG2 and Hep3B cells (Figure 2B, 2C). The knockdown efficiency of si-SBF2-AS1-1 was higher than that of si-SBF2-AS1-3, thus they were used in the following experiment [12]. After knocking down lncRNA SBF2-AS1, the level of miR-361-5p was upregulated in both HepG2 and Hep3B cells (Figure 2D, 2E). The real-time PCR results were in accordance with the prediction results, whereby lncRNA SBF2-AS1 negatively regulated the level of miR-361-5p.

### miR-361-5p negatively regulated the level of TGF-β1 in HCC cells

**Figure 3:**
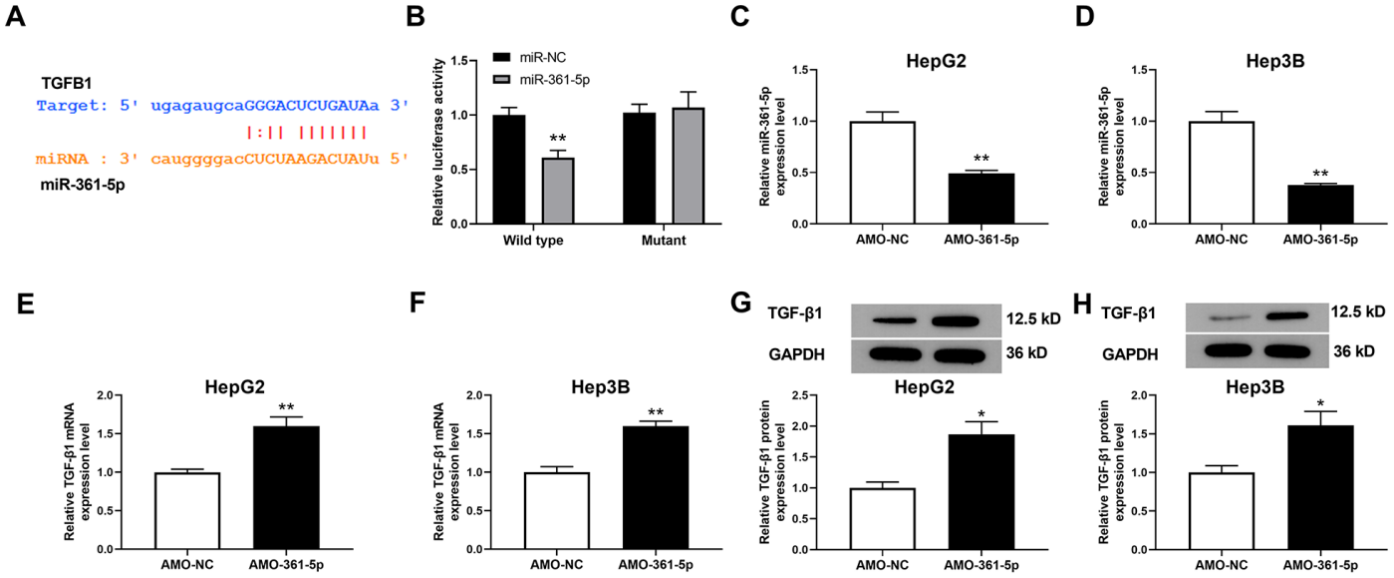
**Relationship between miR-361-5p and TGF-β1.** (**A**) Prediction of the binding site of miR-361-5p and TGF-β1. (**B**) Luciferase reporter assay verified the direct binding effect between miR-361-5p and TGF-β1. (**C**) The effect of AMO-361-5p on miR-361-5p in HepG2 cells. (**D**) The influence of AMO-361-5p on miR-361-5p in Hep3B cells. (**E**) The influence of miR-361-5p knockdown on TGF-β1 mRNA levels in HepG2 cells. (**F**) The influence of miR-361-5p knockdown downregulation on TGF-β1 mRNA levels in Hep3B cells. (**G**) The influence of miR-361-5p knockdown on TGF-β1 protein levels in HepG2 cells. (**H**) The influence of miR-361-5p knockdown downregulation on TGF-β1 protein expression in Hep3B cells. The obtained data were expressed as mean ± SEM. * P < 0.05, ** P < 0.01 vs. si-NC; n=3.

To evaluate miR-361-5p’s downstream molecular output, we predicted its target using ENCORI (http://starbase.sysu.edu.cn/index.php). The prediction results showed that TGFB1 (which encodes TGF-β1) is an object gene of miR-361-5p (Figure 3A). Moreover, the luciferase assay results showed that TGF-β1 directly interacted with miR-361-5p (Figure 3B). AMO-361-5p was used to downregulate the level of miR-361-5p (Figure 3C, 3D). After knockdown of miR-361-5p, the level of TGF-β1 mRNA was upregulated in HCC cells (Figure 3E, 3F). Correspondingly, the downregulation of miR-361-5p also induced the upregulation of the level of TGF-β1 protein (Figure 3G, 3H). These results indicated that TGF-β1 is directly interacts with miR-361-5p.

### Downregulation of miR-361-5p attenuated the influence of lncRNA SBF2-AS1 downregulation on the vitality of HCC cells

**Figure 4:**
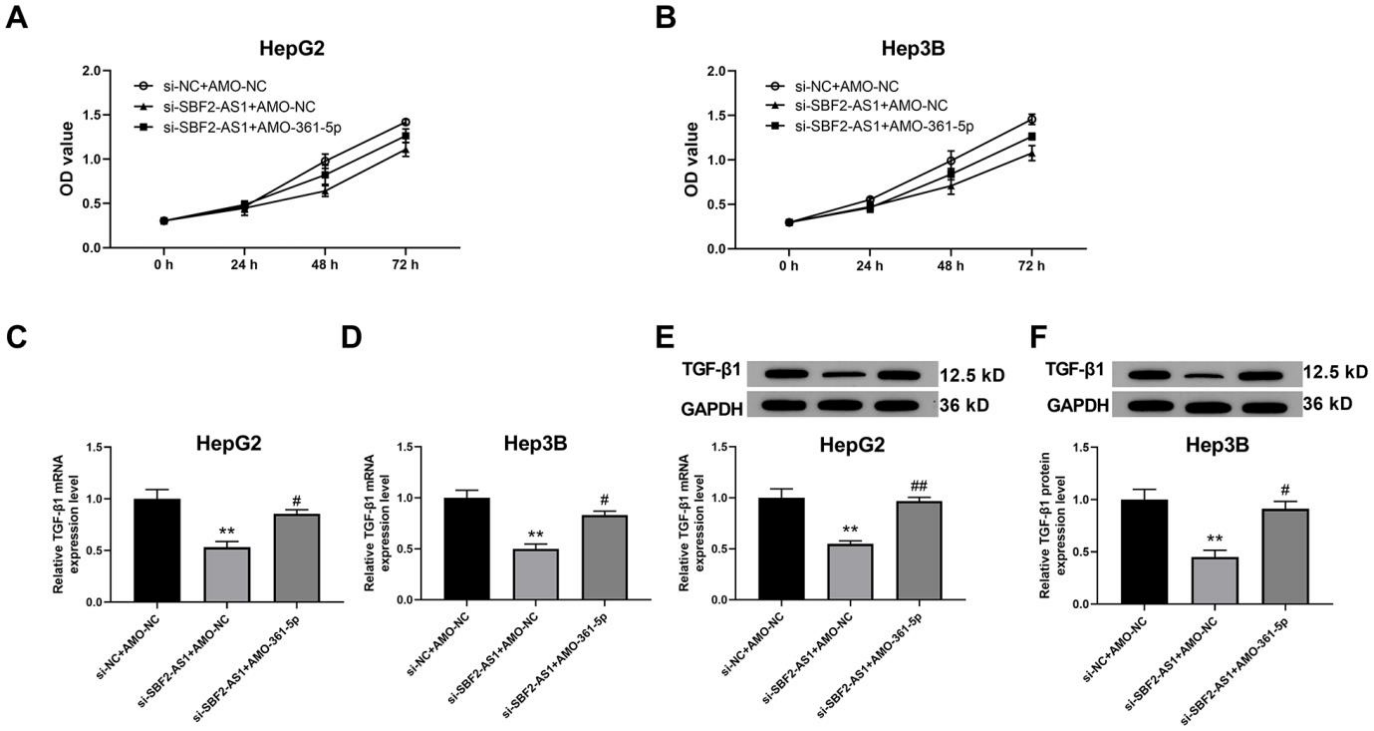
**Suppression of miR-361-5p attenuates the influence of lncRNA SBF2-AS1 downregulation on the viability of HCC cells.** (**A**) The cell viability of HepG2 cells. (**B**) The cell viability of Hep3B cells. (**C**) The mRNA expression of TGF-β1 in HepG2 cells. (**D**) The level of TGF-β1 mRNA in Hep3B cells. (**E**) The level of TGF-β1 protein in HepG2 cells. (**F**) The level of TGF-β1 protein in Hep3B cells. The obtained data are expressed as mean ± SEM. ** P < 0.05 vs. si-NC+AMO-NC; # P < 0.05, ## P < 0.01 vs. si-SBF2-AS1+ AMO-NC; n=3.

The aforementioned results suggested that lncRNA SBF2-AS1/miR-361-5p/TGF-β1 signaling pathway coordination exists in HCC cells. We then explored whether this signaling pathway is involved in the multiplication and migration of HCC tissues. Viability, also an indicator of proliferation of HCC cells, was determined using the CCK-8 kits. The data indicated that the downregulation of lncRNA SBF2-AS1 inhibited the viability of HCC cells in both cell lines (Figure 4A, 4B). Subsequently, we explored the downstream factor expression of this signaling pathway and discovered that knocking down of lncRNA SBF2-AS1 significantly inhibited TGF-β1 expression at both the mRNA and protein level (Figure 4C–4F). Meanwhile, co-administration of AMO-361-5p could downregulate miR-361-5p expression and attenuated the influence of lncRNA SBF2-AS1 (Figure 4).

### Downregulation of miR-361-5p attenuated the influence of lncRNA SBF2-AS1 downregulation on the multiplication of HCC cells

**Figure 5:**
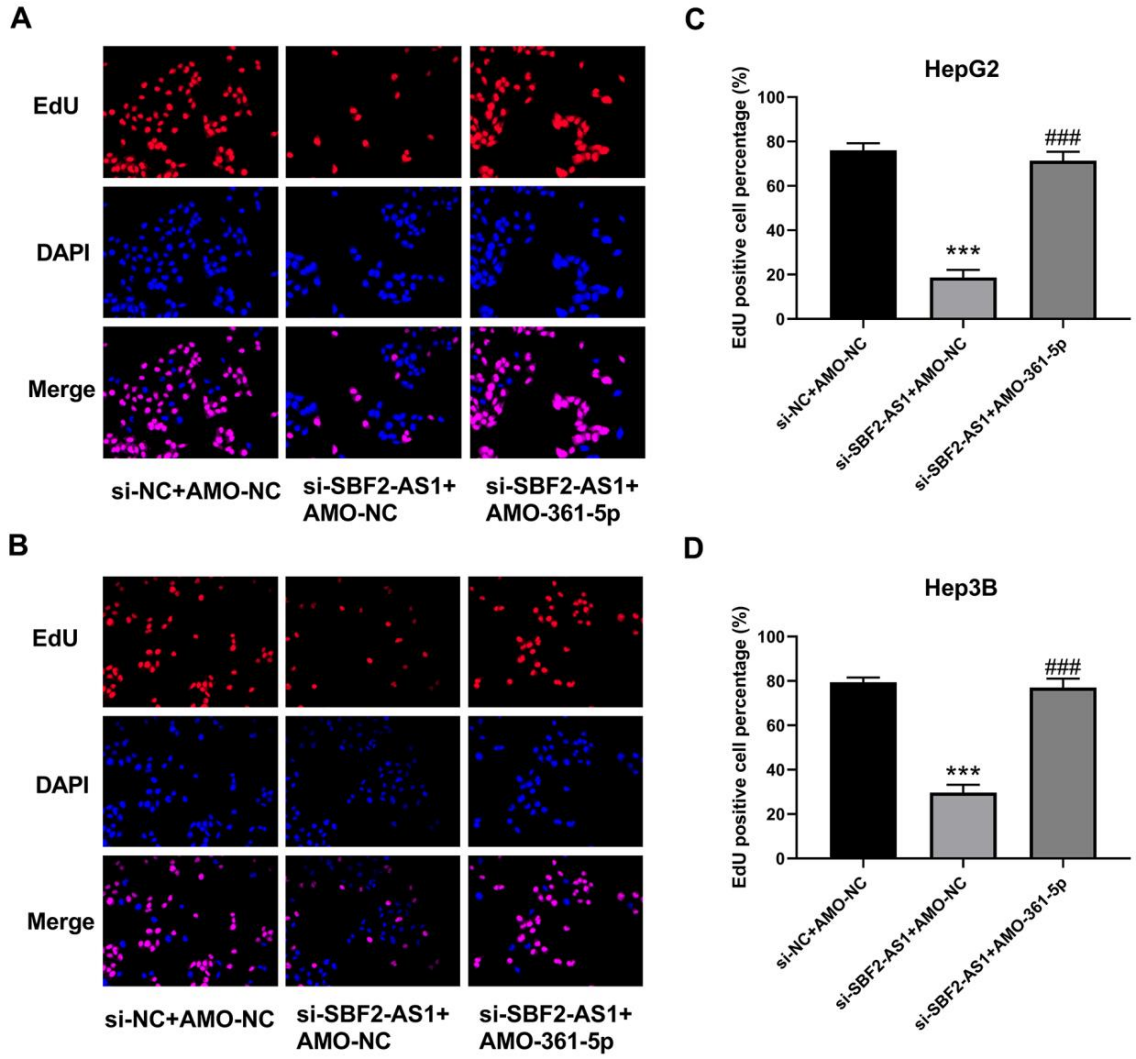
**Suppression of miR-361-5p attenuates the influence of lncRNA SBF2-AS1 downregulation on the proliferation of HCC cells.** (**A**) Representative EdU staining images of HepG2 cells. (**B**) Representative EdU staining images of Hep3B cells. (**C**) The percentage of EdU-positive HepG2 cells. (**D**) The percentage of EdU-positive Hep3B cells. The obtained data are expressed as mean ± SEM. *** P < 0.05 vs. si-NC+AMO-NC; ### P < 0.001 vs. si-SBF2-AS1+ AMO-NC; n=3.

Cell proliferation was evaluated by EdU staining. EdU staining images and the relevant calculated data indicated that the downregulation of lncRNA SBF2-AS1 inhibited the multiplication of both HepG2 and Hep3B cells (Figure 5A–5D). Simultaneously, the downregulation of miR-361-5p attenuated the influence of lncRNA SBF2-AS1 downregulation (Figure 5). Therefore, lncRNA SBF2-AS1/miR-361-5p/TGF-β signaling pathway coordination was involved in the multiplication of HCC cells.

### Downregulation of lncRNA SBF2-AS1 inhibited the transfer of HCC cells

**Figure 6:**
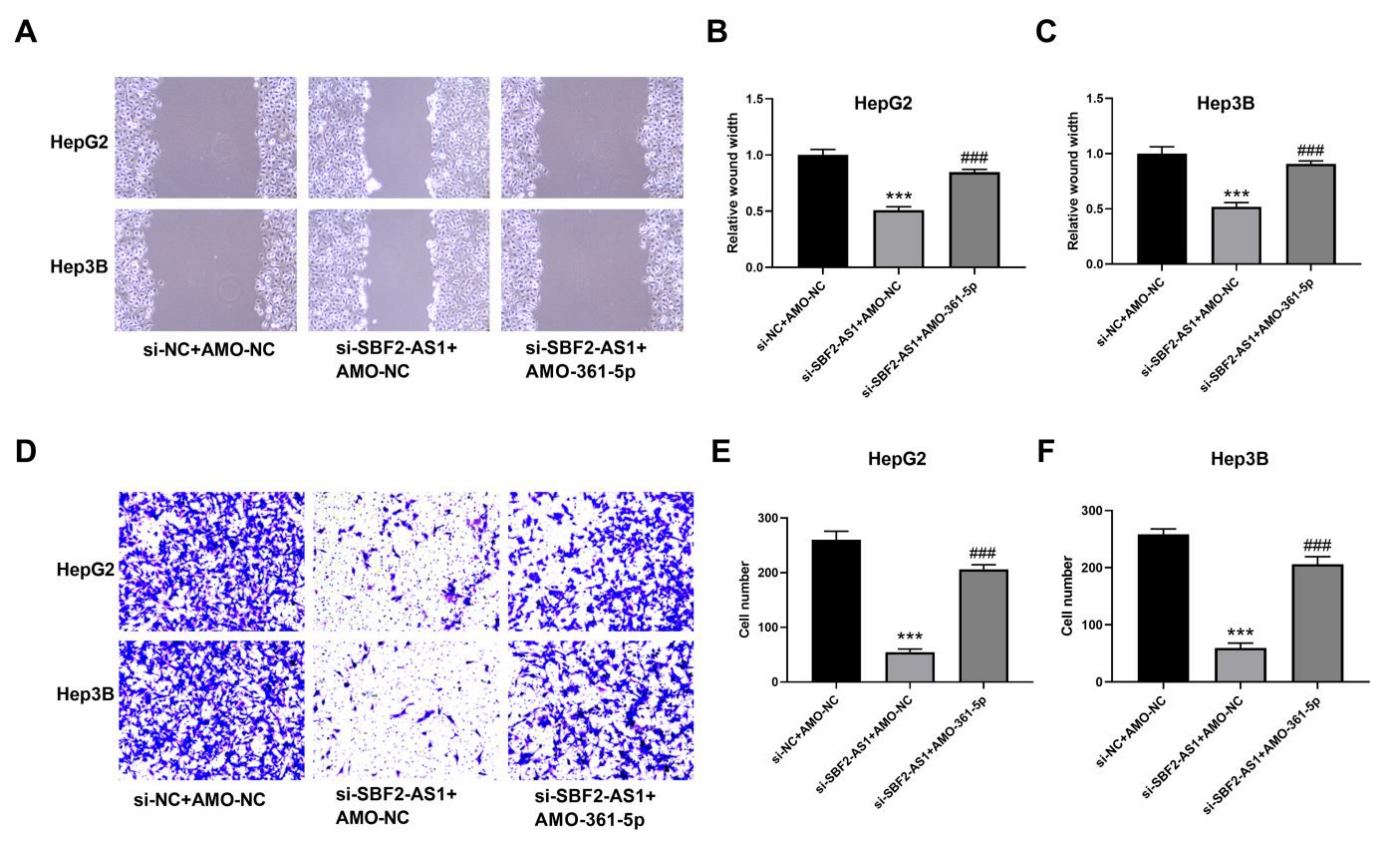
**Suppression of miR-361-5p attenuates the influence of lncRNA SBF2-AS1 downregulation on the migration of HCC cells.** (**A**) Representative images of HepG2 and Hep3B cells. (**B**) Migration distance of HepG2 cells. (**C**) Cell migration distance of Hep3B cells. (**D**) Representative BCCMY images of HepG2 and Hep3B cells. (**E**) The number of migrating HepG2 cells. (**F**) The number of migrating HepG2 cells. *** P < 0.001 vs. si-NC+AMO-NC; ### P < 0.001 vs. si-SBF2-AS1+ AMO-NC; (**A**–**C**), n=6; (**D**–**F**), n=3.

**Figure 7:**
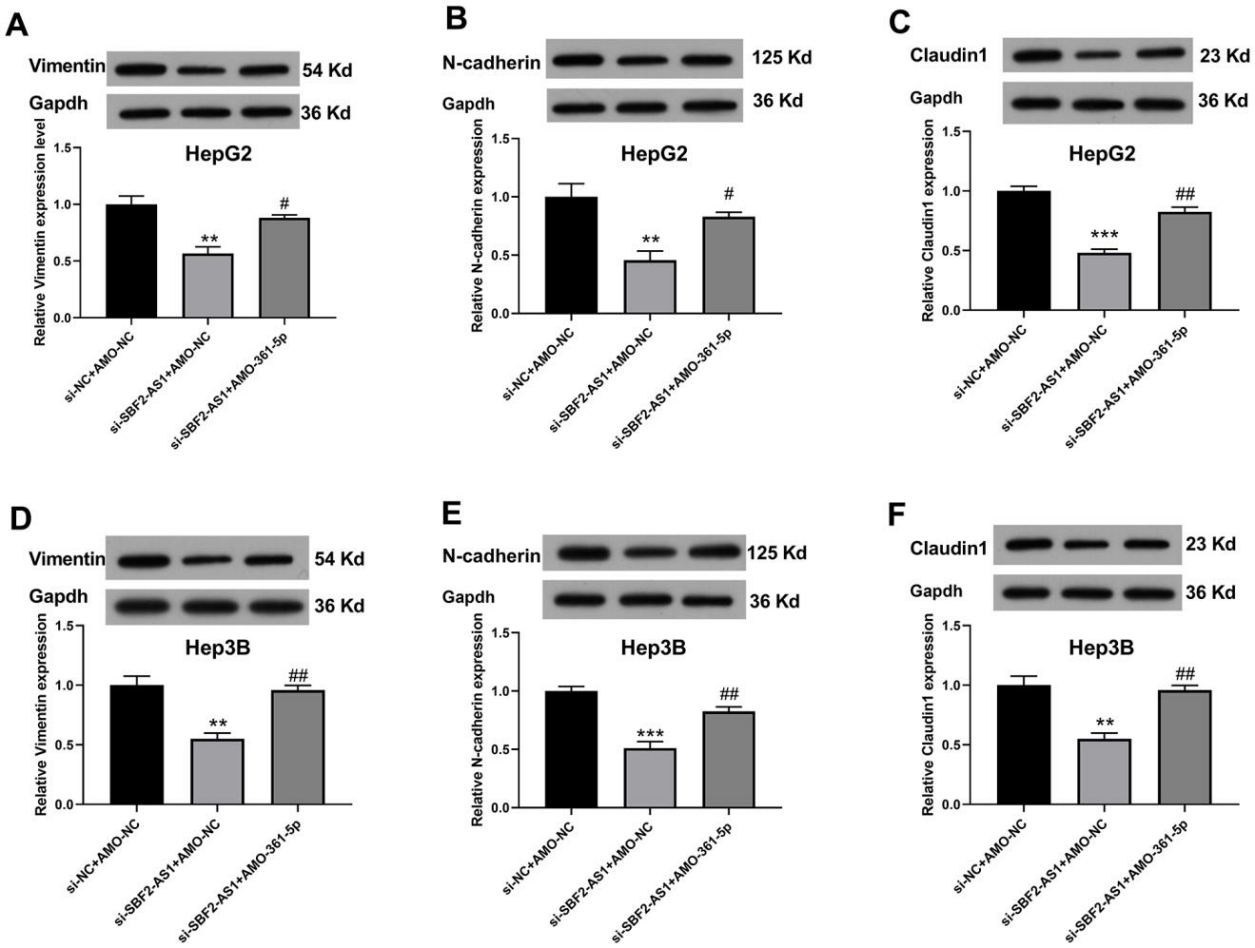
**Suppression of miR-361-5p attenuates the influence of lncRNA SBF2-AS1 downregulation on vimentin, E-cadherin, and claudin-1 protein expression in HCC cells.** (**A**–**C**) The protein expression of Vimentin (**A**), E-cadherin (**B**), and Claudin-1 (**C**) in HepG2 cells. (**D**–**F**) The protein expression of Vimentin (**D**), E-cadherin (**E**), and Claudin-1 (**F**) in Hep3B cells. The data are expressed as mean ± SEM. ** P < 0.01, *** P < 0.001 vs. si-NC+AMO-NC; ## P < 0.01, ### P < 0.001 vs. si-SBF2-AS1+ AMO-NC; n=3.

The migration of HCC cells was measured using WHAY and Boyden chamber cell migration assay. The WHAY results showed that mitigating cell viability was reduced after lncRNA SBF2-AS1 knockdown (Figure 6A–6C). Further, we achieved similar results using BCCMY (Figure 6D–6F). Meanwhile, the co-inhibition of miR-361-5p promoted transfer of HepG2 and Hep3B cells (Figure 6). Therefore, the lncRNA SBF2-AS1/miR-361-5p/TGF-β signaling pathway was involved in the migration of HCC cells. Furthermore, we detected Vimentin, E-cadherin, and Claudin-1 expression. The inhibition of SBF2-AS1 inhibited Vimentin, E-cadherin, and Claudin-1 protein expression, which was attenuated by coadministration of AMO-361-5p (Figure 7A–7F).

### Downregulation of lncRNA SBF2-AS1 inhibited tumor growth in an XMM of HCC

**Figure 8:**
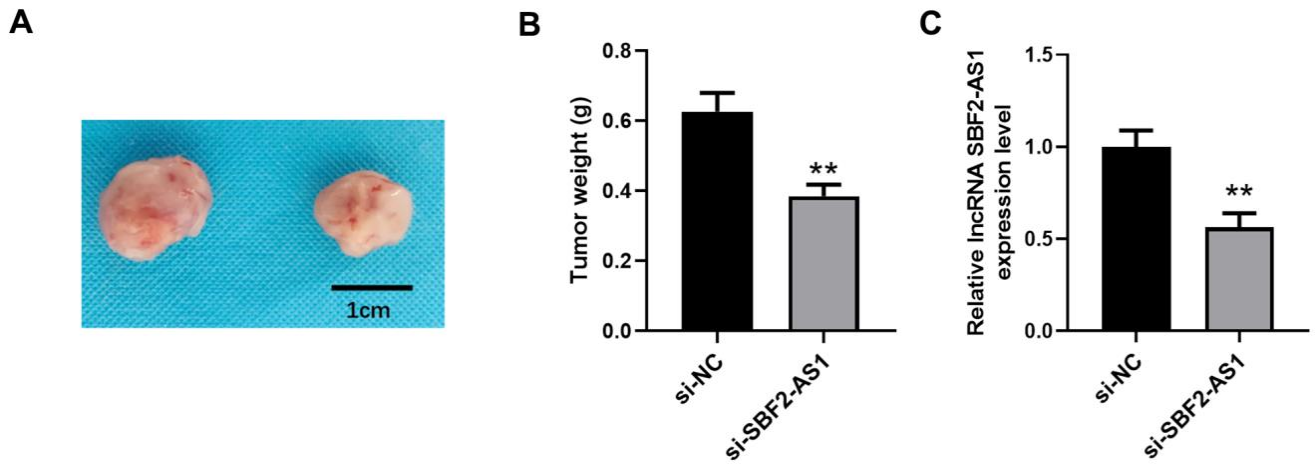
**Downregulation of lncRNA SBF2-AS1 inhibits HCC growth in an XMM.** (**A**) Representative tumor images. (**B**) Statistical results surrounding tumor volume. (**C**) The level of lncRNA SBF2-AS1 in HCC tissues. The obtained data are expressed as mean ± SEM. ** P < 0.01 vs. si-NC; n=6.

We established an XMM using HepG2 cells for the further detection of lncRNA SBF2-AS1 downregulation in non-small cell lung carcinoma (NSCLC). Using a lentivirus, we downregulated lncRNA SBF2-AS1 expression. The data indicated that downregulation of lncRNA SBF2-AS1 considerably inhibited tumor growth (Figure 8A, 8B). Correspondingly, the level of lncRNA SBF2-AS1’ was lower in the si-SBF2-AS1 set compared with that in the si-NC set. Therefore, the downregulation of lncRNA SBF2-AS1 had an inhibitory effect on HCC in an XMM.

### Downregulation of lncRNA SBF2-AS1 upregulated miR-361-5p, while it downregulated TGF-β1 expression in an XMM of HCC

**Figure 9:**
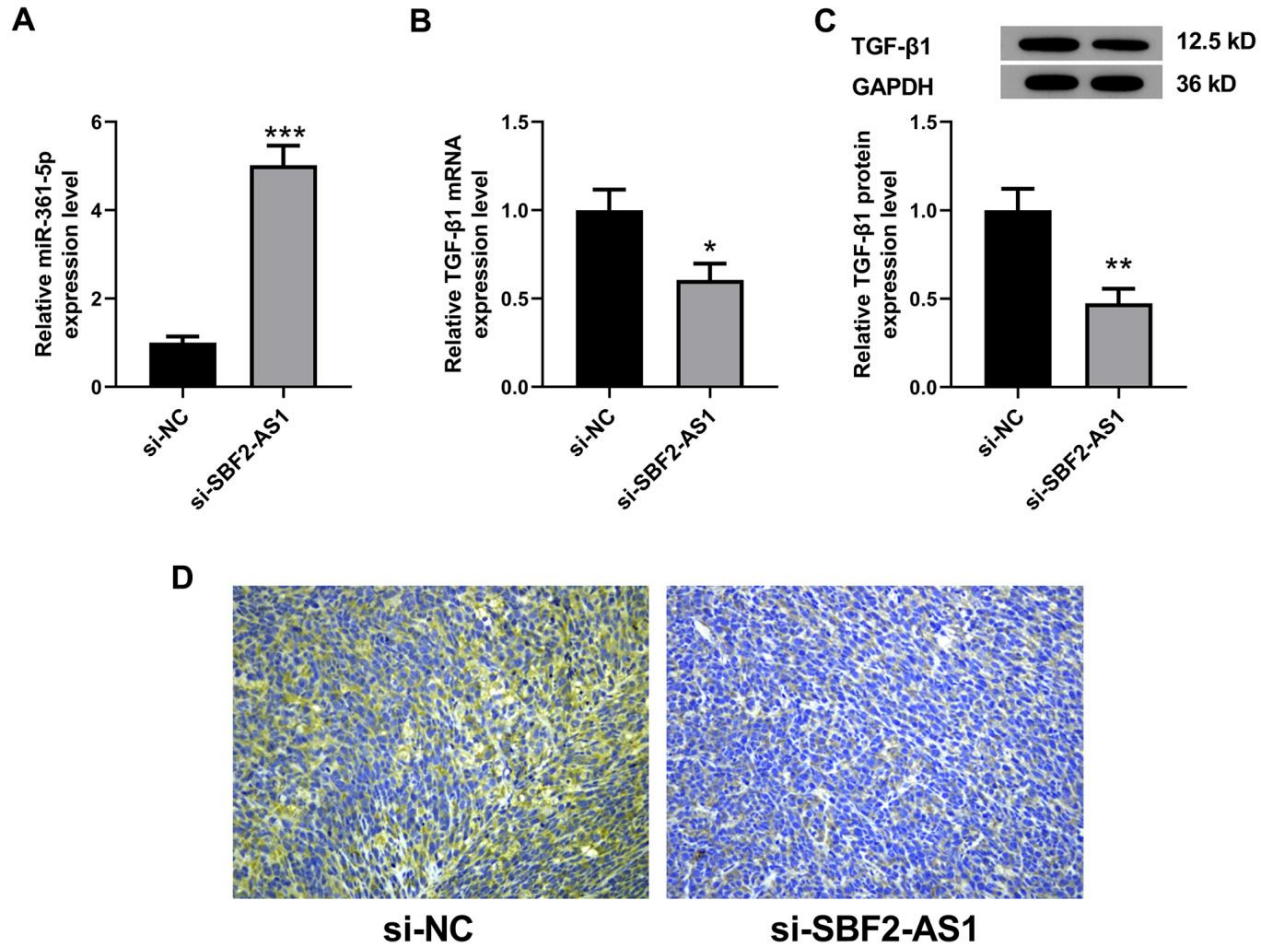
**Levels of miR-361-5p and TGF-β1 after lncRNA SBF2-AS1 knockdown in an XMM of HCC.** (**A**) The level of miR-361-5p in HCC tissues. (**B**) The level of TGF-β1 mRNA in HCC tissues. (**C**) The level of TGF-β1 protein in HCC tissues. (**D**) The representative immunohistochemistry images of TGF-β1. The obtained data are expressed through mean ± SEM. * P < 0.05, ** P < 0.01, *** P < 0.001 vs. si-NC; n=6.

In an *in vitro* experiment, we identified that the lncRNA SBF2-AS1/miR-361-5p/ TGF-β1 signaling pathway played roles in proliferation and migration of HCC cells. Therefore, we further detected the levels of miR-361-5p and TGF-β1 in HCC tissues after lncRNA SBF2-AS1 knockdown. The results showed that miR-361-5p in the si-SBF2-AS1 set is higher in concentration than that in the si-NC set (Figure 9A). Correspondingly, TGF-β1 was downregulated at both the RNA and protein level (Figure 9B–9D). The data shows that the downregulation of lncRNA SBF2-AS1 upregulated miR-361-5p while downregulating TGF-β1 expression in an XMM of HCC.

## DISCUSSION

HCC is still a major cause of death worldwide. In recent years, non-coding RNAs have emerged as an important regulator of the pathogenesis of HCC and demonstrated therapeutic potential [13–15]. However, the reaction and fundamental mechanisms of most differentially expressed lncRNAs are still unknown. In our study, we investigated the effect on and potential mechanism of lncRNA SBF2-AS1 underlying proliferation and migration of HCC cells. The study indicated that the effect of lncRNA SBF2-AS1 was related to the regulation of the miR-361-5p/TGF-β1 signaling pathway.

Emerging research has provided evidence that lncRNAs play important roles in HCC. lncRNA SBF2-AS1 is a functional lncRNA related to different types of cancers [16, 17].

LncRNA SBF2-AS1 is highly expressed in HCC tissues compared with that in adjacent non-tumor tissues, which is also negatively correlated with overall survival of HCC patients [10]. Our research has found that the level of lncRNA SBF2-AS1 was also elevated in HCC cells, which is in accordance with previous research [10].

We predicted the miRNAs that could bind to lncRNA SBF2-AS1 for the further determination of the mechanism of lncRNA SBF2-AS1.

MiR-361-5p was downregulated in HCC tissues and cell lines. The enhanced expression of miR-361-5p inhibit proliferation, invasion and EMT of HCC cells, thus abrogate tumorigenesis [18, 19]. Our data showed that lncRNA SBF2-AS1 and miR-361-5p have binding sites. In addition, the inhibition of lncRNA SBF2-AS1 upregulated the level of lncRNA SBF2-AS1 both in HepG2 and Hep3B cells. In accordance with our results, the interaction between miR-361-5p and lncRNA SBF2-AS1 was also identified in cervical cancer cells [12].

We also predicted that TGF-β1 is the effector gene of miR-361-5p. The downregulation of miR-361-5p upregulated the level of mRNA and protein expression of TGF-β1. The luciferase assay results demonstrated that miR-361-5p could connect to the 3’-untranslated region (UTR) of TGF-β1 directly.

Subsequently, we investigated whether the alteration of miR-361-5p expression could impact the function of lncRNA SBF2-AS1. The data indicated that the inhibition of miR-361-5p attenuated the influence of lncRNA SBF2-AS1 downregulation on HCC cell vitality, proliferation, and migration ability. At the same time, the expression of TGF-β1 was increase after the inhibition of miR-361-5p.

It has been established that miR-361-5p has an inhibitory function in cancer cells. One study suggested that the level of miR-361-5p was downregulated in HCC. Furthermore, the enhanced expression of miR-361-5p inhibits hyperplasia and invasion of HCC cells [18]. Moreover, miR-361-5p could also have that same effect in colorectal cancer, gastric cancer, prostate cancer, etc. [20, 21].

In the present study, TGF-β1 was a target of miR-361-5p. Circulating TGF-β1 levels were considerably higher in HCC patients than patients with other liver diseases [22]. Additionally, circulating TGF-β1 was associated with the staging scores of HCC patients [23].

Moreover, TGF-β1 levels in HCC cells were considerably higher than those in regular hepatic tissues. TGF-β1 was negatively correlated with embryonic liver fodrin. More specifically, these two proteins were associated with disease-free survival and overall survival rates of HCC patients, potentially serving as reliable prognosis biomarkers [24]. In addition, TGF-β1 could stimulate EMT and malignant progression of HCC [25].

We also found that the down regulation of lncRNA SBF2-AS1 also inhibited EMT-related protein expression (Vimentin, E-cadherin, and Claudin-1), which was attenuated by coadministration of AMO-361-5p. These results are in accordance with previous findings, that the inhibition of lncRNA SBF2-AS1 markedly reduced N-cadherin and Vimentin expression [10]. And Claudin-1 could act as a EMT promoter [26].

In other kind of cancers, lncRNA SBF2-AS1 was also shown to act as an onco-lncRNA through sponging miR-302a, miR-143, miR-619-5p, etc [16, 27, 28]. Whether these miRNAs also involved in the effect of lncRNA SBF2-AS1 on HCC still need further investigation.

In conclusion, the downregulation of lncRNA SBF2-AS1 inhibited the multiplication and transfer of HCC cells, which was correlated with the regulation of the miR-361-5p/TGF-β1 signaling pathway.

## MATERIALS AND METHODS

### Cell culture

HepG2 and Hep3B cell lines (from human HCC) and a normal hepatocyte cell line, Bel-7402, were obtained from the Shanghai Institute of Biochemistry and Cell Biology (SIBCB, Shanghai, China). These cells were all stored in RPMI-1640 along with 1% streptomycin/penicillin and 10% FBS. Next, the cells were cultured in a humidifying chamber containing 5% CO_2_ at 37° C.

### Cell proliferation assay

We used a CCK-8 (Beyotime, Shanghai, China) assay kit to detect the viability of cells. The experiment was carried out with rigid application of the manufacturer’s instructions.

### Wound healing assay (WHAY)

HCC cells were inoculated in a 6-well culture plate and scratched with a germfree 10-μL pipette tip. Then, the culture medium was removed and the plates were washed three times with the medium. The width of the open area was measured before and 48 h after treatment. The distance of the wound was measured and calculated [29].

### Boyden chamber cell migration assay (BCCMY)

After 24 hours of berberine pre-treatment, the cells were inoculated in serum-free medium at a rate of 1.5×104 cells/well, and then cultured for 24 hours at 37° C. We used methanol to fasten the migrated cells and Giemsa to stain. Further, we utilized a light microscope to count the number of cells.

### EdU staining

The influence of lncRNA-SBF2-AS1 on cell proliferation was tested by EdU staining. HCC cells were inoculated on a 6-well plate, then transfected in the expected order. Forty-eight hours after treatment, the Cell-Light TM EdU assay (RiboBio, Guangzhou, China) was conducted on the basis of the manufacturer’s protocol to evaluate the proliferation of HCC cells.

### Xenograft mouse model (XMM) establishment

Six-week-old male BALB/c nude mice were procured from the Shanghai Laboratory Animal Company (Shanghai, China). The mice were raised in a pathogen-free environment, and they received standard residual tooth animal food *ad libitum*. The HepG2 cells in serum-free culture medium (5×10^6^/100 μL) were injected into the back of the mice subcutaneously [30]. Lentivirus carrying the sequence against lncRNA SBF2-AS1 was administered to mice to knockdown lncRNA SBF2-AS1, while negative control mice received lentivirus with a negative control sequence. After treatment for 21 days, the mice were sacrificed and tumor tissues were dissected for further analysis. All applicable international, national, and/or institutional guidelines for the care and use of animals were followed.

### Western blot assay

We calculated total protein extracted from the cells or tissues using western blotting. After the protein was loaded onto the wells, SDS-PAGE was carried out, following which the membrane was transferred to a nitrocellulose filter by electrical transfer. Then, the membrane was blocked for 2 hours in 5% fat-free milk. Thereafter, the membranes were incubated with primary antibodies including TGF-β1 (cat: 3711; 1:1000), Vimentin (cat: 5741; 1:500), E-cadherin(cat: 3195; 1:500) and Claudin-1 (cat: 4933; 1:1000) from Cell Signaling Technology (MA, USA), alone with GAPDH (cat: TA-08; 1:2000; ZSGB-BIO, Beijing, China) at 4° C overnight. Thereafter, we incubated the membranes with secondary antibody for 1 hour at room temperature. Finally, the bands on the membrane were quantified.

### Real-time PCR assay

For SBF2-AS1 location detection, subcellar fractions were separated by NE-PER Nuclear and Cytoplasmic Extraction Reagents (Thermo, Scientific, MA, USA). For other experiment, total RNA in HCC cells or tissues was determined by TRIzol reagent (Roche, IN, USA). Then, 500 ng of RNA was prepared to create the cDNA based on the corresponding instructions of the expression kit (Toyobo, Osaka, Japan). GAPDH or U6 were used as internal references. Real-time PCR was performed and quantified according to the 2^-ΔΔCT^ method. The primer sequences are as follows. SBF2-AS1: forward 5’-CACGACCCAGAAGGAGTCTAC-3’, reverse 5’-CCCGGTACCTTCCTGTCATA-3’; TGFB1: forward 5′-GGATACCAACTATTGCTTCAGCTCC-3′, reverse 5′-AGGCTCCAAATATAGGGGCAGGGTC-3′; miR-361-5p: forward 5′-TAGCTTATCAGAATCTCCAGGGG-3′, reverse 5′-CAGAATCACACCTGGGGGAC-3′; GAPDH: forward 5’-GCACCGTCAAGGCTGAGAAC-3’, reverse 5’-TGGTGAAGACGCCAGTGGA-3; U6: forward 5’-CTCGCTTCGGCAGCACA-3’, reverse 5’-AACGCTTCACGAATTTGCGT-3’.

### Cell transfection

Corresponding negative controls (si-NC) and small interfering RNA targeting lncRNA SBF2-AS1 (si-SBF-AS1) were obtained from RiboBio (Shanghai, China). Anti-miR-361-5p oligonucleotides (AMO-361-5p), as well as its isotype controls (AMO-IC), were obtained from RiBoBio (Shanghai, China). The X-treme GENE siRNA transfection reagent (Roche, Basel, Switzerland) was used to transfect sequences into cells.

### Luciferase reporter assay

HepG2 cells were inoculated on 12-well plates. Then, the plasmid was constructed and transfected into those cells. The luciferase count was standardized using Tk-Renilla-luciferase (Promega, USA). The activity of luciferase was analyzed by luminometer and a dual-luciferase assay kit (Promega, USA).

### Data analysis

The obtained data were expressed as the mean ± SEM. Statistical comparisons between different groups or among multiple groups were identified by Student's t-test or ANOVA, respectively. The analysis of the percentage difference was carried out by the χ^2^ test. Statistical significance was determined when there was a *p* value of less than 0.05.
